# Small Rotations, Big Effects: Lessons from Water Adsorption in NU-1000

**DOI:** 10.1021/acs.jpcc.4c06889

**Published:** 2025-02-05

**Authors:** Filip Formalik, Bartosz Mazur, Faramarz Joodaki, Bogdan Kuchta, Randall Q. Snurr

**Affiliations:** † Department of Chemical and Biological Engineering, Northwestern University, Evanston, Illinois 60208, United States; ‡ Department of Micro, Nano and Biomedical Engineering, Faculty of Chemistry, Wroclaw University of Science and Technology, 50-370 Wroclaw, Poland

## Abstract

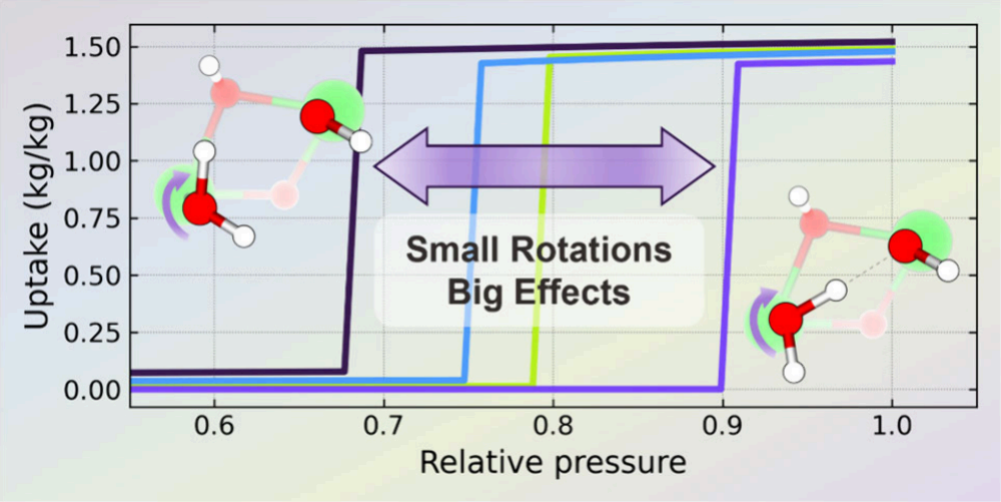

In this study, the adsorption mechanism of water in the metal–organic framework NU-1000 was investigated using molecular simulations. The simulations predict a significant impact of small changes in terminal aquo ligand orientation on the shape and pressure of the condensation step in the water adsorption isotherm. The analysis revealed that the rotational mobility of aquo ligands, often neglected in computational studies, can shift the condensation step by up to 20% in the relative humidity scale. By examining adsorption modes and interaction sites, it was demonstrated that configurational changes in the Zr_6_O_8_ node affect water adsorption significantly and can change the nature of the interactions from hydrophobic to hydrophilic. We propose a robust approach to account for these changes in simulations, achieving good agreement with experimental results. This work underscores the necessity of considering local, molecular flexibility in water adsorption simulations to avoid mischaracterization of MOFs’ water adsorption properties.

## Introduction

Water harvesting is an important potential application of metal–organic frameworks (MOFs) and covalent organic frameworks (COFs).^1−4^ This process utilizes significant temperature differences, which can be created using some kind of energy or which occur naturally, such as in day-night cycles. These natural cycles are particularly pronounced in dry environments like deserts. At night, cooler temperatures facilitate the adsorption of water, while during the day, higher temperatures aid in its desorption, using solar heat as the energy source. This approach was shown to work even in extremely dry environments such as Death Valley National Park or the Mojave Desert, where the average relative humidity is approximately 14%. A device designed in the group of Omar Yaghi was shown to operate in such conditions and produce between 210 and 285 g (about 1 cup) of water per kilogram of porous material per day.^5^ This process has the potential to solve the problem of water scarcity in such places as sub-Saharan regions.^6^

MOFs that could be considered for water harvesting applications must naturally be characterized by high water stability. One of the most promising water harvesting materials is MOF-303. It is built with 1D Al(OH) inorganic chains and pyrazole-based linkers.^7^ The polarity of the linkers promotes the formation of a water hydrogen bond network that facilitates a condensation step at relative humidities as low as 10% with a full saturation at 20%, which was confirmed by in situ single crystal X-ray diffraction studies and Gibbs ensemble Monte Carlo (GEMC) simulations.^8^ It was also shown that further modification of this MOF, such as linker extension, can increase its working capacity and decrease the operation costs.^9,10^ Another group of MOFs that exhibit outstanding water stability are frameworks with Zr_6_O_8_ nodes. Microporous frameworks such as MOF-801 and MOF-841 have shown good performance in water adsorption with 22.5 and 44 wt % uptake at 10% and 30% relative humidity (RH), respectively.^11−13^ MOF-808 with larger pores (about 20 Å) shows even higher capacity at a slightly larger RH of 36%.^14^

Although efforts based on chemical intuition or trial and error searches to find new structures applicable to water harvesting have proven successful, they can be time-consuming, and it is challenging to systematically correlate the properties of the best-performing structures. Computational methods have emerged as valuable tools for elucidating physical and chemical mechanisms of water adsorption in MOFs, facilitating a targeted search for new materials with exceptional performance.^15−22^ In particular, in the past decade, screening studies have become a powerful tool for the identification and computational characterization of MOFs for various properties. Nevertheless, only one report in the current literature explores high-throughput computational screening to identify best-performing MOFs for water harvesting.^23^

**Figure 1:**
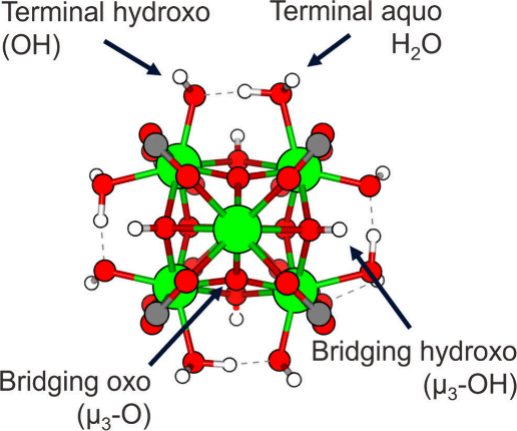
Topology of the Zr_6_O_8_ node, as used in this study, follows the structure proposed by Planas et al.^24^ It distinguishes between two types of oxygen-containing ligands: bridging and terminal. Bridging oxo and hydroxo ligands coordinate with three neighboring Zr ions, while terminal hydroxo and aquo ligands coordinate with a single Zr ion. This configuration minimizes the Gibbs free energy of the node and ensures its neutrality, which is not always the case in structures that only incorporate terminal hydroxo or bridging oxo ligands, as sometimes seen in the literature.

In this work, we focus on the adsorption mechanism of water in the Zr-MOF NU-1000. Through a detailed description of the adsorption process at low relative humidities, we show that small changes in the orientation of terminal aquo ligands on the Zr_6_O_8_ node (see Figure 1 for visualization of the node) can shift the condensation step by as much as 20% in RH scale, although these small changes are almost always neglected in computational adsorption studies. First, we analyze the adsorption modes and interaction sites for a water molecule in the environment of the Zr_6_O_8_ node. We then analyze the impact of the changes in the ligand topology of the node on the condensation step within the adsorption isotherm and provide a molecular-level analysis of the condensation process. Finally, we present a simple but robust approach that accounts for the configurational changes of the Zr_6_O_8_ node and show that with its use, one can obtain very good agreement with experimental results.

## Methods

### Cluster DFT Calculations

Cluster calculations were performed with Gaussian 16, Rev. C03^25^ with the M06-L/def2-TZVP^26−28^ level of theory adopted for all atoms, in addition to the def2-TZVP/ECP for Zr atoms. The Grimme empirical dispersion correction D3^29^ was applied to account for long-range dispersion interactions. Thermochemical analysis was performed based on vibrational partition functions obtained from analytical frequency calculations at 298 K and 1 atm.

### Complexation Energy and Enthalpy of Adsorption

The complexation energy was calculated as
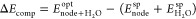
where *E*_node+H_2_O_^opt^ is the electronic energy of the optimized node with adsorbed water and *E*_node_^sp^ and *E*_H_2_O_^sp^ are the single point energies of the node and water molecule at the same geometry as in the complex. The energies were corrected for basis set superposition error. The enthalpy of adsorption was calculated as
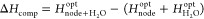
where *H*_node+H_2_O_^opt^ is the enthalpy of the optimized node with adsorbed water and *H*_node_^opt^ and *H*_H_2_O_^opt^ are enthalpies of the optimized bare node and an optimized (isolated) water molecule. The individual enthalpies were calculated as the sum of translational, rotational and vibrational internal thermal energies:
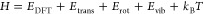


Enthalpies were calculated using the GoodVibes software^30^ and account for the frequency scaling factor 0.982 proposed by Truhlar et al.^31^

### Periodic DFT Calculations

Periodic structures of the NU-1000 variants in this work were optimized with periodic DFT as implemented in VASP 5.4.4 package with the PAW method.^32−34^ We applied the cutoff energy for the plane-wave-basis set as 700 eV. Both the unit cell parameters and ionic positions were allowed to change in the geometry optimization procedure. Energy convergence was 10^–6^ eV for the SCF cycles, and the force convergence was set to 0.01 eV/Å for geometry optimization steps. The PBE^35^ density functional with D3(BJ)^29,36^ empirical dispersion correction was applied for geometry optimization calculations. The DDEC6 method^37^ was used for partial charge calculations, as implemented in the chargemol software.^38^

### Adsorption Simulations

In this work, we utilized the single-macrostate implementation of the TMMC method,^39^ also called NVT + ghost swap method, with the interpolation scheme reported in our recent work.^40^ All simulations were performed using an in-house modification of the RASPA2 code.^41^ Simulations were not performed for every possible value of *N*. Instead, we performed simulations for *N* = 0, 10, 20, . . . ,*N*_max_, and missing data were generated by linear interpolation between known values of the transition probability matrix. Each simulation was performed for at least 50 000 initialization cycles and 100 000 production cycles, where a cycle is defined as max(20, *N*). Convergence was monitored based on the ratio of rate of acceptances for molecule insertions versus deletions, where a simulation is considered to be converged when this ratio remains constant. Water molecules were modeled with the TIP4P model.^42^ NU-1000 was modeled as rigid with a combination of Lennard-Jones (LJ) parameters from UFF^43^ for Zr atoms and Dreiding^44^ for remaining atoms. The Lorentz–Berthelot mixing rules were employed to describe LJ interaction parameters for unlike interactions. Interactions were truncated at a spherical cutoff of 12.8 Å, and no analytical tail corrections were applied. For electrostatic interactions, we utilized the Ewald summation. Partial charges for framework atoms were calculated with the DDEC6 method as described above.

To employ the reweighing method, the fugacity coefficient was kept constant during the simulation at a value of 1 so that the fugacity is equal in value to the pressure set in the simulation input. During the isotherm calculation, the true pressure was calculated by mapping the fugacity to the pressure of the bulk fluid of the same water model and simulation parameters, with eight independent WL/TMMC simulations^45^ using the FEASST software.^46^ The values of saturation pressure and the saturation fugacity coefficient are presented in Table S1. For more information on the theory behind the TMMC simulations and reproducibility of the results generated in this work, see the **SI**.

## Results & Discussion

**Figure 2:**
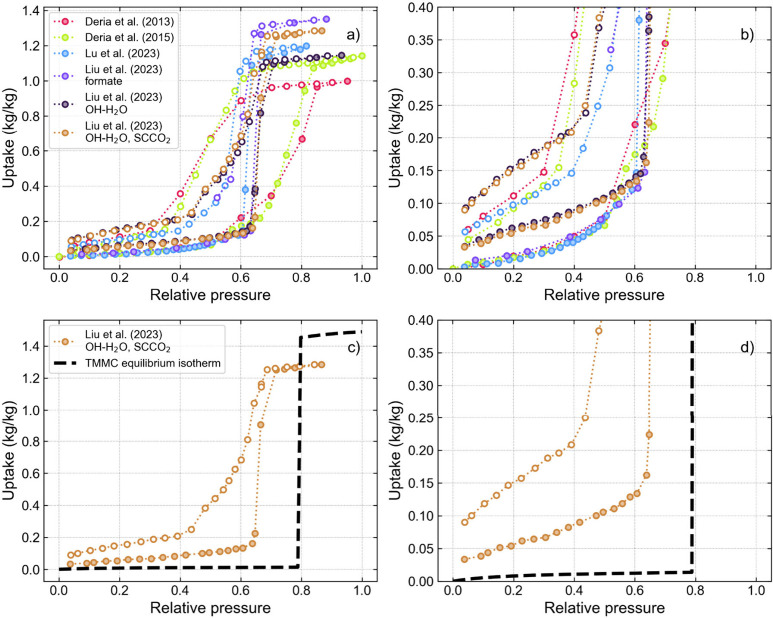
(a) Experimental water isotherms in NU-1000 MOF, measured at 298 K, from various literature reports^22,47−49^ (data points digitized from published figures) (b) with a zoom-in to the low-uptake range. The three isotherms presented in a) from Liu et al. correspond to the structures with formate modulators and OH-H_2_O ligands. The structure indicated as OH-H_2_O was activated using a standard dehydration approach, and the structure indicated with SCCO_2_ was activated with supercritical carbon dioxide. (c) The Liu OH-H_2_O ligands experimental isotherm is compared with the TMMC continuous isotherm from simulations, obtained using the “standard” NU-1000 structure, d) with a zoom-in to the low-uptake range. The filled and open symbols represent adsorption and desorption data points, respectively.

When performing molecular simulations, it is a common practice to validate the model by comparing the results with experiment. In Figure 2a and b we compiled experimental isotherms of water in NU-1000 from various literature reports.^22,47−49^ There are noticeable discrepancies: at a low RH of 4%, uptake on the adsorption branch of the isotherm varies from 0.001 to 0.035 kg/kg, and the saturation uptake ranges from 1.00 to 1.35 kg/kg. The RH of the condensation step ranges from about 60% to 80%, and the width of the hysteresis loop varies significantly from approximately 5% to 30% RH. We hypothesize that the differences in uptake at low RH may be due to chemical variations in the Zr_6_O_8_ nodes across different samples, while the variations in saturation uptake may be attributed to residual molecules in the pores or partial pore collapse. It is known from recent literature that the chemical composition of the node depends on the synthesis procedure. Lu et al. showed that NU-1000, which has an 8-connected node (i.e., 8 linkers are attached to the node), can have at least three different types of modulators or ligands coordinated to the remaining 4 sites of the node: formate, two aquo ligands with a halogen anion or an aquo-hydroxo pair.^50^ This diversity can naturally make the node more hydrophobic (in the case of formate) or hydrophilic (in the case of the other two terminations). Some earlier literature did not report the nature of the modulators or ligands on the nodes of NU-1000, which makes it difficult to compare simulation and experiment. However, a recent study by Liu et al.^22^ reported water adsorption isotherms for carefully characterized samples with a clear distinction among the three different possible terminations, and thus we decided to use their data for comparison in this study. A structural representation of the Zr_6_O_8_ node considered in this work is presented in Figure 1.

In this work, all simulated isotherms were obtained with the NVT + ghost swap variant of the transition matrix Monte Carlo (TMMC) method,^51−53^ which is part of the family of flat histogram methods.^54−56^ Thus, all presented isotherms represent true grand canonical isotherms (or equilibrium isotherms),^57^ where the step is always observed at the binodal point (pressure at which low-density and high-density states have the same free energy and hence are in equilibrium). The isotherms are continuous because the histogram reweighting method allows for achieving infinite pressure/fugacity resolution. For the simulations, we used an in-house modification^41^ of the RASPA2 software^58^ and a recently proposed method of interpolation between the macrostate numbers.^40^ For more details on the methodology, see the SI. Since the crystallographic structure of NU-1000 lacks hydrogen atoms on the Zr_6_O_8_ node, we generated the structure with OH-H_2_O ligands manually following the proton topology with the lowest free energy, as suggested by Planas et al.^24^ Different topologies are sometimes used in the literature, like hydroxo–hydroxo pairs,^59^ but these represent an inaccurate chemical composition of the node with an unbalanced charge and are, thus, unphysical. The structure of NU-1000 with the correct node topology was optimized with periodic density functional theory (DFT) calculations (we refer to this structure as *standard*), and the partial charges were subsequently generated for the adsorption simulations (see SI).

**Figure 3:**
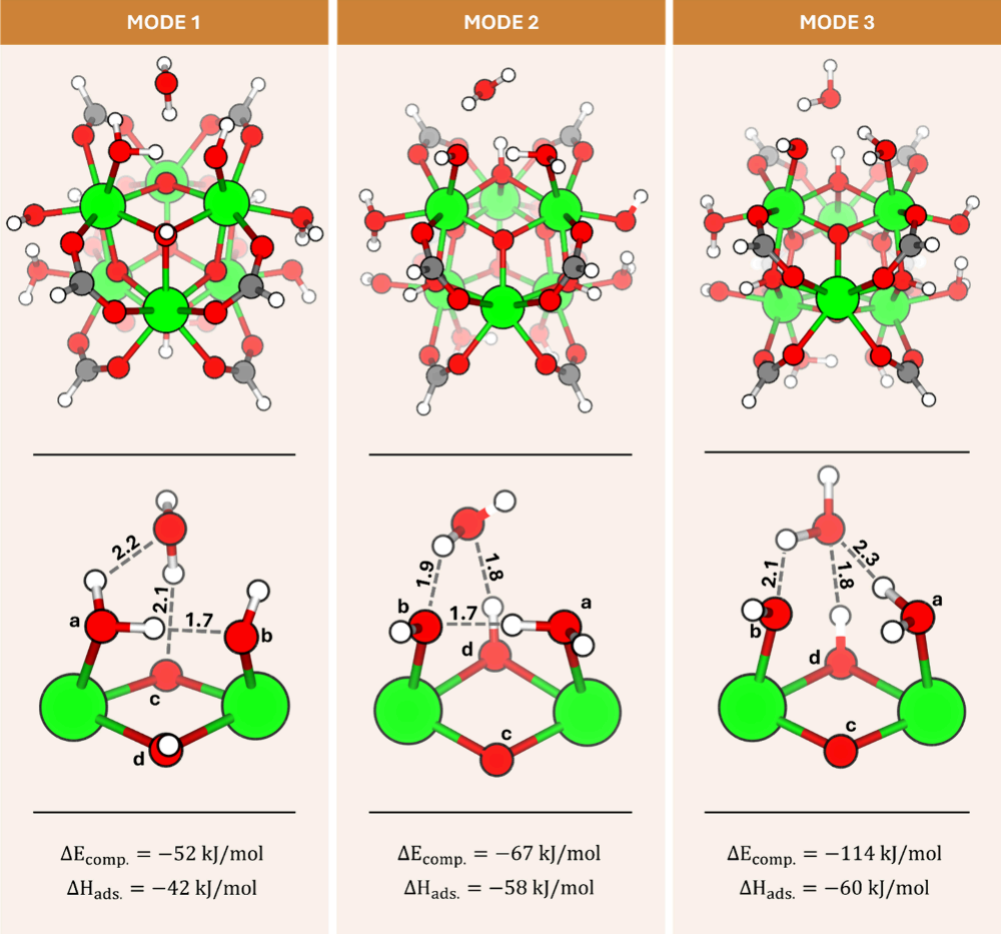
Water adsorption modes on the Zr_6_O_8_ node obtained from DFT structure optimization. At the top, the figure illustrates the node configuration with the first adsorbed water molecule. The middle section presents a zoom-in to a portion of the cluster at the adsorption site. The letters represent (a) terminal aquo, (b) terminal hydroxo, (c) bridging oxo, (d) bridging hydroxo ligands. The bond lengths are provided in units of Å. At the bottom, both the complexation energy and the enthalpy of adsorption are presented. White, gray, red, and green colors of atoms represent H, C, O, and Zr, respectively.

The simulated adsorption isotherm obtained with a standard structure of NU-1000 is presented in Figure 2c and d. It is apparent that the simulation results do not match the corresponding experimental isotherm in any of the key descriptors (condensation step RH, saturation loading, and shape of the isotherm before the step). Since the TMMC method guarantees that the isotherm is properly equilibrated, the discrepancy can stem from two factors: inaccurate force field parameters used for simulations and inaccurate structure of the MOF. To investigate this, we analyzed the interaction of water molecules with the Zr_6_O_8_ node using DFT within the localized basis set approximation, using the cluster presented in Figure 3 as the representation of the node. A detailed presentation of the types of bridging and terminal ligands within the node of NU-1000 is shown in Figure 1. We found three distinct adsorption modes of water adsorbed on the node. To characterize them we use the energy of complexation (which reflects the strength of interactions between cluster and adsorbed water) and the enthalpy of adsorption (which includes thermal energy and captures the effect of reorganization of atoms after adsorption, for more details we refer to the Methods section). In mode 1, the water molecule adsorbs adjacent to the bridging oxo (μ_3_O, c) ligand of the node, with a hydrogen bond being formed between this oxygen and a hydrogen atom of the adsorbed water molecule (distance of 2.1 Å). This water molecule also forms a hydrogen bond with the terminal aquo ligand (a) that coordinates to the Zr acid site on the node (2.2 Å). In mode 2, we observe a very similar configuration, but the adsorbed water now forms a hydrogen bond with the bridging hydroxo ligand (μ_3_OH, d), which is slightly more exposed compared to the bridging oxo (the distance between the central bridging oxygen atom and a plane formed by three adjacent zirconium atoms is 0.4 Å in case of μ_3_O and 1.0 Å for μ_3_OH), facilitating formation of a stronger (shorter) hydrogen bond at a distance of 1.8 Å. The second hydrogen bond is formed with the oxygen from the terminal hydroxo ligand (1.9 Å, b) (versus with the terminal aquo in mode 1). These shorter and hence stronger hydrogen bonds between adsorbed water and the node in mode 2 are reflected in a higher (magnitude) complexation energy and enthalpy of adsorption (mode 1: Δ*E*_comp_ = −52 kJ/mol and Δ*H*_ads_ = −42 kJ/mol; mode 2: Δ*E*_comp_ = −67 kJ/mol and Δ*H*_ads_ = −58 kJ/mol). In both modes 1 and 2, a third, strong, intranode hydrogen bond is formed between the terminal aquo and hydroxo ligands (1.7 Å). The hydrogen bond angles in the studied clusters span a range of 130° to 180°, with the atomic coordinates for each cluster provided in the **SI** (as XYZ files).

The third water adsorption mode represents a seemingly small difference in the node configuration, which, however, significantly impacts the energy of interactions. The adsorbed water molecule forms three hydrogen bonds: one with the hydrogen atom of a bridging hydroxo ligand (d), serving as the hydrogen bond acceptor; another with the hydrogen atom of a terminal aquo ligand (a), also serving as the hydrogen bond acceptor; and a third with the oxygen atom of a terminal hydroxo ligand (b), as the hydrogen bond donor. This configuration contrasts with the formation of only two hydrogen bonds in modes 1 and 2. It is reflected in an almost 2-fold increase in the complexation energy (−114 kJ/mol). The enthalpy of adsorption increases only slightly due to an energy penalty associated with the rotation of the terminal aquo (by approximately 45°), which involves breaking the intranode hydrogen bond (between terminal hydroxo and aquo, as present in modes 1 and 2). We emphasize that except for the rotation of the terminal aquo ligand and breaking of the intranode hydrogen bond, the configuration of the node does not change. This shows that small changes in the configuration of atoms in the Zr_6_O_8_ node can significantly affect the potential energy surface for molecules absorbing within its close environment.

**Figure 4:**
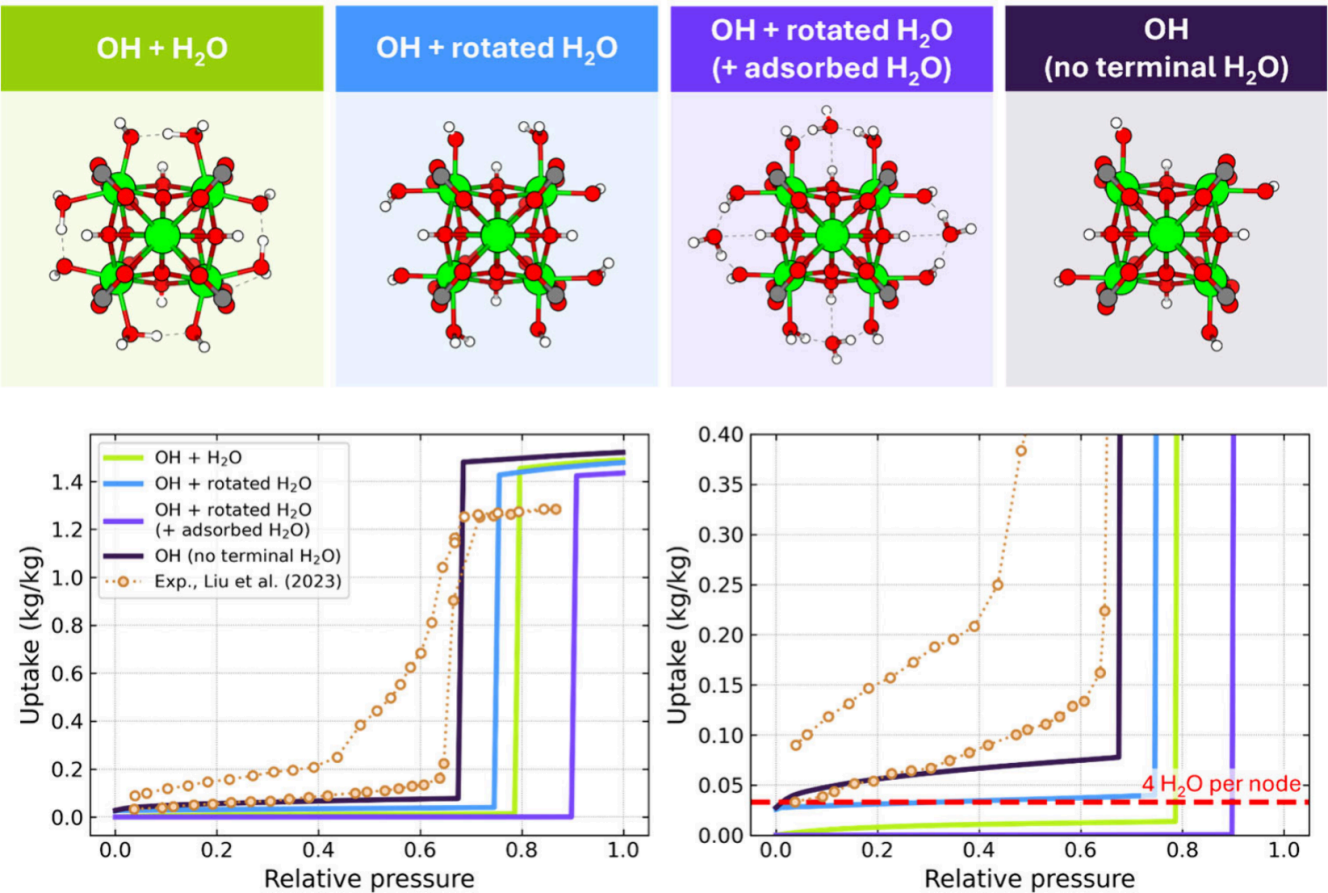
Zr_6_O_8_ nodes of NU-1000 structural variants used in simulations (top) and corresponding TMMC simulated isotherms compared to experiment (bottom) at 298 K.

To examine how these changes impact the adsorption process, we generated new structures of NU-1000 with varying configurations of terminal hydroxo and aqua groups on the nodes, using periodic DFT calculations (see SI for methodological details). Beyond the standard model (black isotherm in Figure 2c and d, described as OH + H_2_O structure), where the structure of the MOF is optimized without any guest water molecules to resemble configurations observed in modes 1 and 2 (with the intranode hydrogen bond), we included the first adsorbed water in the optimization procedure to enforce the rotation of the terminal aqua group, mimicking mode 3. This yielded two additional structures: in the first, we retained the first adsorbed water to analyze its influence on the adsorption of further molecules (OH + rotated H_2_O + adsorbed H_2_O); and in the second, this water molecule was removed after optimization, leaving a node with a rotated terminal aquo ligand (OH + rotated H_2_O). The Zr_6_O_8_ nodes of these two NU-1000 structural variants are shown together with the standard structure of the node in Figure 4 with the corresponding adsorption isotherms. Using the green isotherm (representing the standard model) as a reference, we observe that when the terminal aquo ligand is rotated (blue isotherm), the formation of the hydrogen bonds between subsequent molecules is promoted, the precondensation uptake shifts upward by approximately 4 molecules per node, and the condensation step shifts toward lower pressure (i.e., toward the pressure at which the condensation step occurs in the experimental isotherm) by 4% RH. While this change is modest, it indicates that even a minor alteration in the node’s configurational environment can affect the adsorption mechanism. In contrast, when the first adsorbed water is present in simulations (and the entire node, including this molecule, is kept rigid, purple isotherm), the uptake prior to condensation is negligible and the condensation step is shifted to 90% RH. This shows that imposed rigidity in the adsorbent during adsorption simulations can artificially render the MOF hydrophobic, as the adsorption site environment cannot adjust to form a hydrogen bond network.

With this analysis in mind, we decided to take a step toward reducing the rigidity constraints imposed on the adsorption site environment of the node. To achieve this, we completely removed the terminal aquo ligand, allowing water to adsorb naturally during simulations and to settle into a configuration that minimizes the energy and facilitates the formation of hydrogen bonds. This creates an open Zr site that attracts water molecules,^60^ and we denote the structure as OH without terminal H_2_O. This approach is not an artificial computational trick but is motivated by the fact that the Zr nodes lose their aquo ligands when treated thermally between 60 and 120 °C.^61^ Moreover, prior to adsorption experiments, samples are typically activated at such temperatures,^22^ suggesting that this structure reflects the possible configuration of real samples. The corresponding isotherm, presented in Figure 4 (dark purple), shows that when water is allowed to adsorb naturally on the open Zr site during adsorption simulations, the simulated isotherm approaches the experimental data points. The condensation step is now present at 68% RH, and, equally importantly, the precondensation part of the isotherm exhibits a more hydrophilic character, resembling the experimental curve. Furthermore, even at very low pressures, the uptake is approximately 4 molecules per node. Simulation snapshots confirm that the initial adsorption sites for water are now the open Zr sites. Unlike in the scenario where water is considered part of a rigid framework, water molecules on the Zr sites can now rotate and adjust freely to either form intranode hydrogen bonds or facilitate the formation of hydrogen bonds with other adsorbed water molecules. In Figure S4, based on TMMC simulations snapshots, we show that the configuration of the node changes depending on the number of water molecules in the system, adopting configurations that reflect different modes predicted with DFT geometry optimization.

To connect the calculations with the hysteresis observed in the experimental isotherm, we utilized TMMC to determine the spinodal points, which define the stability limits of the adsorbed phases. Although the spinodals do not directly tell us whether or where hysteresis, which is a kinetic phenomenon, might occur, they outline the possible hysteresis range. Figure S6 shows isotherms with the metastable branches of both the low- and high-density phases. Our results reveal that the pressure range between the spinodals is much larger than the experimental hysteresis, with the low-density branch extending beyond the saturation pressure. The experimental hysteresis is narrow, with the condensation and evaporation steps occurring at very close pressures. Thus, in Figures 2 and 4 we used the equilibrium isotherm from TMMC as the representative quantity to compare with experiment.

To enable a direct comparison of the simulated isotherms with experimental data, we calculated the bulk saturation pressure of water using TMMC bulk phase calculations with the same water model (same parameters, cutoff distance and lack of the tail correction) used in the adsorption simulations. Although the saturation pressures differ between experimental and simulated data, plotting the results on a relative scale (*P/P*_sat_) allows for a meaningful comparison^62,63^ (for details on saturation pressure calculations, see methodology in SI, specifically Table S1).

**Figure 5:**
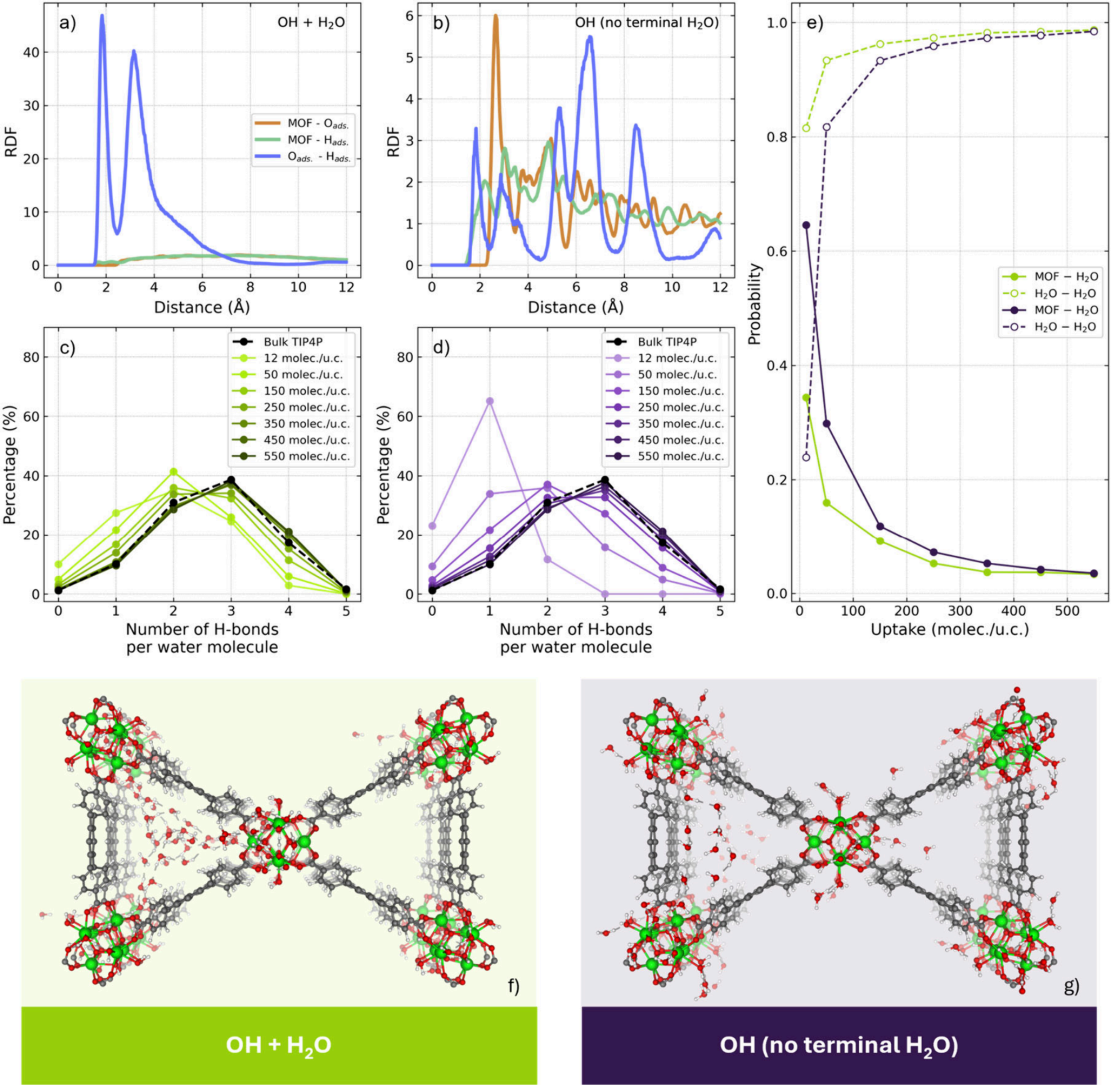
Differences in adsorbed water behavior at a loading of 12 water molecules per unit cell in two configurational variants of NU-1000: standard (OH + H_2_O) and modified (OH with no terminal H_2_O): RDF (a and b), hydrogen bond analysis (c, d and e) and visual representation (32 molecules per unit cell snapshot) of water clustering versus adsorbing on the pore walls (f and g).

To further analyze the configurations of water molecules during adsorption, we examined the radial distribution functions (RDFs) shown in Figure 5 and S2, considering both MOF-water and water–water distances (for the MOF, all atoms are considered). The results were obtained from a simulation containing 12 water molecules per unit cell (4 molecules per node). The MOF-adsorbate RDFs remain nearly flat for both the standard model and the model where the first adsorbed water molecule is rigidly integrated into the framework. In these models, almost all interactions are between adsorbate molecules, and hence, water tends to cluster rather than adsorb onto the node, despite its inherently hydrophilic nature due to numerous OH and aquo sites (Figure 5a and S2c). This implies that the rigidity of molecular configurations of the node in the simulations imparts an artificial hydrophobicity to the MOF, altering the shape of the adsorption isotherm and causing the simulation results to deviate from experimental observations significantly. In the modified structure of NU-1000, where the aquo group was specifically rotated to facilitate adsorption, the RDF reveals that the adsorbate molecules interact predominantly with the node (Figure S2b). The last model, with removed terminal aquo ligands, represents the most realistic scenario where adsorbate molecules primarily adsorb onto the open zirconium sites and can form hydrogen bonds with either adjacent hydroxo ligands or additional guest molecules (Figure 5b).

The investigated structural variations of NU-1000 significantly influence the condensation of water within its pores. These structural changes are reflected in the changes of free energy barriers between the low and high-density states of water absorbed in the pores, obtained in TMMC simulations and shown in Figure S3 (see the TMMC simulations methodology for details on free energy calculations). The values range from 95 to 115 kT per unit cell, depending on the structure. This variation indicates that the configurational modifications not only affect the shape of the precondensation isotherm and the position of the condensation step but also facilitate the phase transition.

A quantitative analysis of the hydrogen bonds further supports these findings. Figure 5c and d present the distribution of hydrogen bonds per water molecule in both the standard structure and the modified structure with aquo ligands removed. In the standard structure (Figure 5c), the distribution closely resembles that of bulk water, even at low loadings, suggesting that water forms clusters instead of adsorbing to the pore walls. Conversely, in the modified MOF configuration (Figure 5d), at a low loading of 12 molecules per unit cell, most water molecules form only one hydrogen bond, indicating a stronger interaction with the framework. As the number of molecules increases, this distribution gradually aligns with that of the bulk fluid. Figure 5e shows the probability of a water molecule forming hydrogen bonds with either the MOF or other adsorbed molecules. For the standard NU-1000 structure, the likelihood of bonding with other molecules consistently exceeds that with the MOF. In the structure without terminal aquo ligands, this trend shifts during adsorption: initially, molecules primarily interact with the MOF and later with each other. Such a mechanism was previously shown to be responsible for facilitating the water condensation in the pores of some other MOFs.^21^ Finally, we illustrate the spatial distribution of the water molecules in both structures of NU-1000 discussed above (Figure 5f and g). In the standard structure, water clustering is prominent within the trigonal micropores, whereas in the modified structure (OH with no terminal H_2_O), water is more evenly distributed among adsorption sites and forms smaller clusters. This finding supports the conclusion that the standard structure promotes artificial water clustering in a rigid, hydrophobic environment.

When the aquo ligand is removed from the node and water molecules are allowed to interact directly with the Zr atoms in the Monte Carlo simulations, the coordination bond between Zr and water is modeled using a combination of Lennard-Jones and Coulomb potentials. To assess the appropriateness of this approximation, we compared interaction energies and distances from the force field with those from DFT. Table S2 presents the host–guest interaction energy from the force field for the first water molecule adsorbed on the MOF surface across all four NU-1000 variants. The results indicate that the OH (no terminal H_2_O) shows notably stronger interaction energy (−94.5 kJ/mol), highlighting the ability of the model to capture the strong affinity between the open Zr site and the aquo ligand’s oxygen. While the computed interaction energy is slightly less favorable than the DFT-calculated value (Δ*H*_ads,DFT_ = −126.9 kJ/mol), the simulations accurately capture water’s preferred adsorption site at the Zr open site. We also compared O_H2O_–Zr distances, with DFT values ranging from 2.23–2.31 Å and force field distances slightly larger at 2.42–2.6 Å, both indicating stable configurations near the Zr site. RDFs for a NVT MC simulation with 100 water molecules per unit cell (Figure S5) show a sharp peak at 2.46 Å for the OH (no terminal H_2_O) system, confirming water localization at Zr sites. Tracking O_H2O_–Zr distances during the simulations further supports this, demonstrating consistent retention of water near Zr open sites, while allowing rotational flexibility in response to the local environment. Further improvements in accuracy could involve using the 12–6–4 LJ potential proposed by Leem and Chen,^60^ applying the TraPPE isopropanol force field for terminal and bridging hydroxo ligands, as suggested by Wardzala et al.,^64^ or developing a customized force field that allows for full framework flexibility.

## Conclusions

In conclusion, we demonstrated that beyond large structural dynamics such as breathing or gate opening, MOFs exhibit another level of flexibility that can significantly influence water adsorption,^8^ similar to what has been previously observed with carbon dioxide.^65^ We introduced a model of NU-1000 where the terminal aqua groups are initially removed from the node before adsorption simulations, creating a strong adsorption site. This enhances their ability to adjust their orientation according to the local adsorptive environment. Using novel computational methods for adsorption simulations, we have shown that this approach yields results that are both physically meaningful and in reasonable agreement with experiments. DFT calculations identified three potential adsorption modes of water around the Zr node of NU-1000, indicating that rotation of the terminal aqua group is energetically favored in the presence of adsorbate molecules. Altering the orientation of the terminal aqua group attached to the node can shift the adsorption condensation step on the simulated isotherm by as much as 20%, and the precondensation uptake from 0 to 8 molecules per Zr_6_O_8_ node. By analyzing radial distribution functions, hydrogen bonds, and simulation snapshots, we demonstrated that changes in the isotherms are linked to the artificial hydrophobicity of NU-1000 in the standard (OH + H_2_O) model, commonly used in simulations. Conversely, when the terminal aquo ligand is allowed to move freely, it can adopt a configuration that facilitates the creation of a hydrogen bonding network with MOF and among adsorbate molecules. We anticipate that these conclusions and the computational methodology can be applied to other zirconium-based MOFs and potentially other MOFs where water tends to bind to open metal sites.

## Data Availability

The input and output files used to generate the presented results and figures are available on Zenodo: 10.5281/zenodo.14651142.

## Supporting Information Available

The structure of Zr_6_O_8_ node; N_2_ isotherms and pore size distribution; Methodology and simulation details, RDF for all structures discussed in this work; Free energy profiles (PDF)
Data for the reproduction of the adsorption isotherms from Figure 4 (AIF). Atomic positions of the DFT-optimized configurations from Figure 3 (ZIP)
