# Supplementary material for: Small Rotations, Big Effects: Lessons from Water Adsorption in NU-1000
Source: J Phys Chem C Nanomater Interfaces. 2025 Feb 5;129(7):3752–61. doi: 10.1021/acs.jpcc.4c06889 (PMC11848914; doi:10.1021/acs.jpcc.4c06889)
Supplement: Supplementary file 1 — jp4c06889_si_001.pdf [file jp4c06889_si_001.pdf]

Supporting information for:

Small Rotations, Big Effects: Lessons from Water Adsorption in NU-1000

Filip Formalik[1,2,=], Bartosz Mazur[2,=], Faramarz Joodaki[1], Bogdan Kuchta[2]*, Randall Q. Snurr[1]*

[1]Department of Chemical and Biological Engineering, Northwestern University, Evanston, IL 60208, United States

[2]Department of Micro, Nano and Biomedical Engineering, Faculty of Chemistry, Wroclaw University of Science and Technology, 50-370 Wroclaw, Poland

[*]**Corresponding authors:** snurr@northwestern.edu, bogdan.kuchta@pwr.edu.pl

Characterization of NU-1000

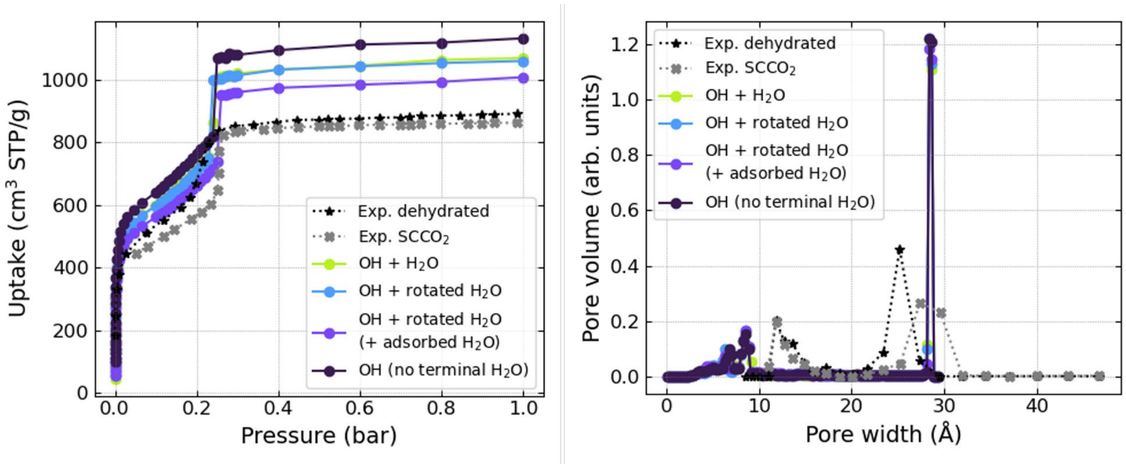

Figure S1. *Comparison of textural properties from experimental data of Liu et al.*[1] *and from our simulations of NU-1000 structures: N_2 isotherms at 77 K (left) and pore size distributions (right). The label "dehydrated" refers to data from structures activated using the standard approach, while "SCCO2" denotes structures activated with supercritical carbon dioxide. Simulated pore size distributions were obtained using PoreBlazer software.*[2]

Transition matrix Monte Carlo simulations

The primary objective of the TMMC method is to calculate the macrostate probability distribution (MPD) instead of directly calculating ensemble averages, as in the conventional GCMC method.[3–6] For simulations of adsorption phenomena, the number of particles in the system, denoted N, can be used as a macrostate variable. The MPD in the grand canonical ensemble Π is related to fundamental statistical mechanical quantities by:

$$\Pi(N;\ \mu, V, \beta) = \frac{\exp(\beta\mu N) Q(N, V, \beta)}{\Xi(\mu, V, \beta)} \qquad (1)$$

where μ is chemical potential, V is system volume, $\beta = 1/k_B T$ (T being temperature and k_B being the Boltzmann constant), Q is canonical partition function, and Ξ is grand canonical partition function. The determination of the MPD is accomplished by updating the so-called collection matrix (C-matrix) with the unbiased probabilities of accepting an insertion or deletion move (p_{acc}) at each visit to a macrostate N. This is done according to the following equations:

$$C(N_o \to N_n) = C(N_o \to N_n) + p_{acc}(o \to n) \qquad (2)$$

$$C(N_o \to N_o) = C(N_o \to N_o) + [1 - p_{acc}(o \to n)] \qquad (3)$$

where the labels o and n refers to *old* and *new* configurations for the attempted move. Next, the transition probability matrix is calculated from the C-matrix:

$$P(N \to N + \delta) = \frac{C(N \to N + \delta)}{\sum_{\delta \in \{-1,0,1\}} C(N \to N + \delta)} \qquad (4)$$

As simulation is restricted only to transitions between adjacent macrostates values of $\delta = -1, 0, +1$. The quantity $\Pi(N;\ \mu, V, \beta)$ can be calculated using the following protocol: first, an arbitrary value assigned to $\ln \Pi(N_{min})$, after which the values of $\ln \Pi$ for the subsequent N are calculated sequentially using the following equation:

$$\ln \Pi(N+1;\ \mu, V, \beta) = \ln \Pi(N;\ \mu, V, \beta) + \ln\left[\frac{P(N \to N+1)}{P(N+1 \to N)}\right] \qquad (5)$$

Finally, $\ln \Pi$ is normalized. The ensemble average of any physical quantity A in the grand canonical ensemble is calculated using the following equation:

$$\langle A_\alpha \rangle = \frac{\sum_{N\in\alpha} A(N, V, T)\Pi(N;\ \mu, V, T)}{\sum_{N\in\alpha} \Pi(N;\ \mu, V, T)} \qquad (6)$$

where the sums are calculated over the macrostates that belong to phase α. The value of interest, denoted by A, is accumulated for each visited macrostate N. The phase boundary is defined as the local minimum in $\ln \Pi$.

The GC-TMMC simulations, performed at a given μ value, can be recalculated to any other μ' value by histogram reweighting, according to the following equation[5]:

$$\ln \Pi(N;\ \mu', V, T) = \ln \Pi(N;\ \mu, V, T) + \ \beta N(\mu' - \mu) \qquad (7)$$

or, in terms of fugacity f:

$$\ln \Pi(N;\ f', V, T) = \ln \Pi(N;\ f, V, T) + N \ln\left(\frac{f'}{f}\right) \qquad (7')$$

This approach enables the derivation of the full isotherm at infinite resolution from a single set of calculations performed at a single μ value without additional simulation effort. The minimum and maximum number of particles should be set to ensure that Π has tails with a sufficiently low probability at the upper and lower limits of the macrostate range. Therefore, we used $N_{min} = 0$, while for N_{max} we checked that Π was sufficiently low after each reweighting using the equation:

$$\max\left[\ln \Pi\left(N_{liquid}\right)\right] - \ln \Pi(N_{max}) > 10 \qquad (8)$$

In practice, Equation 8 indicates that the N_{max} state has a relative probability of e^{-10} with respect to the nearest local minimum. Consequently, it has a negligible effect on the ensemble averages.

The MPD is strictly related to the relative grand potential free energy W of a particular macrostate N MPD (e.g., as shown in **Figure S4**) through[4]:

$$W(N; \mu, V, T) = -kT \ln \Pi(N; \mu, V, T). \qquad (9)$$

For a more detailed description of the identification of phases and the calculation of thermophysical properties from MPD, we refer the reader to the paper by Siderius *et al.*[3]

Table S1. Saturation pressure and fugacity, and fugacity coefficient for the TIP4P model at 298 K with 12.8 Å cutoff for LJ interactions. The error was calculated as the standard deviation of the average of eight independent simulations.

$\boldsymbol{p_{sat}}$ **(Pa)**	**error (Pa)**	$\boldsymbol{f_{sat}}$ **(Pa)**	**error (Pa)**	$\boldsymbol{\varphi = f_{sat}/p_{sat}}$	**error**
4695	15	4596	15	0.9789	0.00035

Reproducibility

All simulations were performed using an in-house modification of the RASPA2 code: https://github.com/b-mazur/RASPA2_GC-TMMC.git.

The ASAF library was used to interpolate the transition matrix and calculate the isotherms: https://github.com/b-mazur/asaf.

To facilitate reproducibility, example simulation files and the modified RASPA code are available via https://github.com/b-mazur/.

Hydrogen bond analysis

We used our in-house C++ package to analyze the number of hydrogen bonds between water molecules and between water molecules and the framework.[7] Two criteria were considered to identify hydrogen bonds: the H···O distance had to be less than 2.5 Å, and the O···H–O angle had to be greater than 150°.[8] Terminal aquo, terminal hydroxo, bridging oxo, and bridging hydroxo ligands of the Zr node were considered for hydrogen bonding calculations. This calculation was applied every 250 cycles for the final 25000 cycles of each simulation.

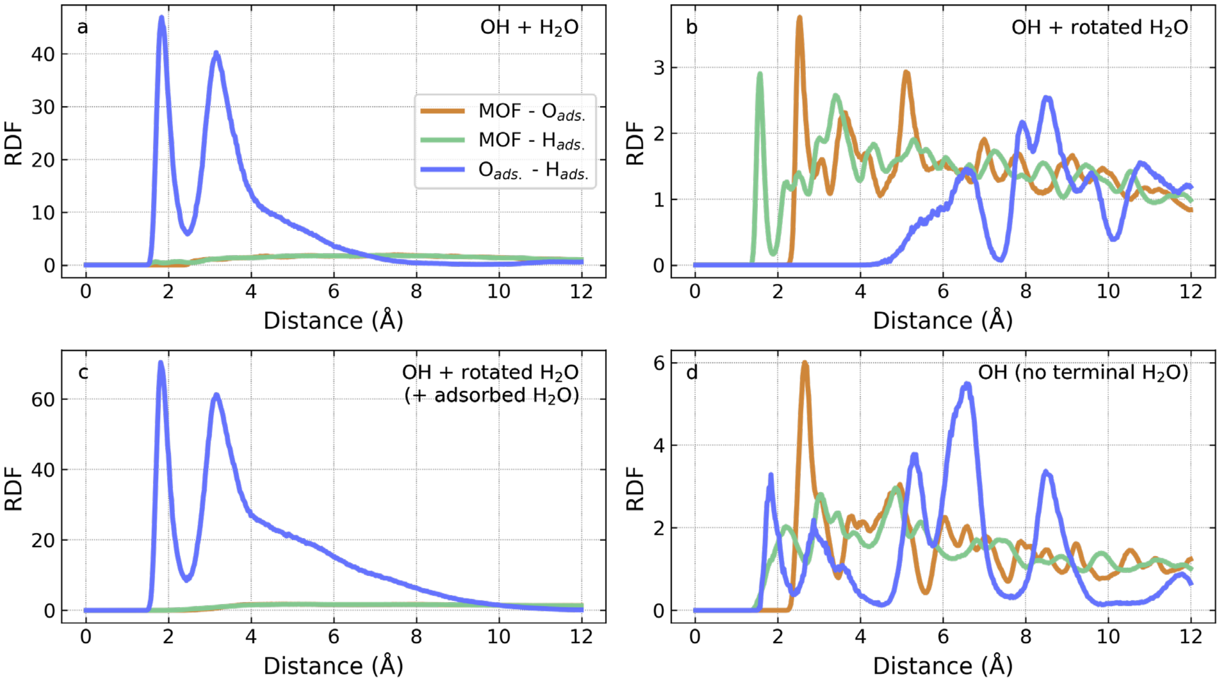

Figure S2. *Radial distribution functions for water in NU-1000 at 298 K for 4 molecules per node loading. The results show the distances for close contacts: MOF - adsorbed water molecules and water – water for all four configurational variants of NU-1000 discussed in this work.*

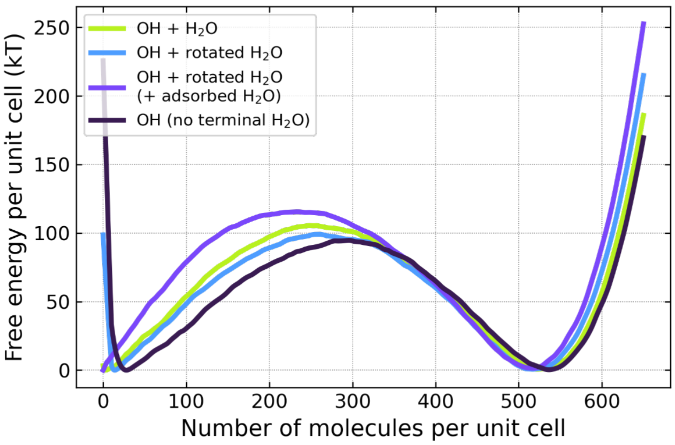

Figure S3. *Free energy profiles showing the barriers between low-density and high-density states at the equilibrium pressure, obtained with **Equation 9.** The barriers are: 105, 99, 115, 95 kT per unit cell for OH + H_2O, OH + rotated H_2O, OH + rotated H_2O (+ adsorbed H_2O) and OH (no terminal H_2O), respectively.*

Configuration comparison

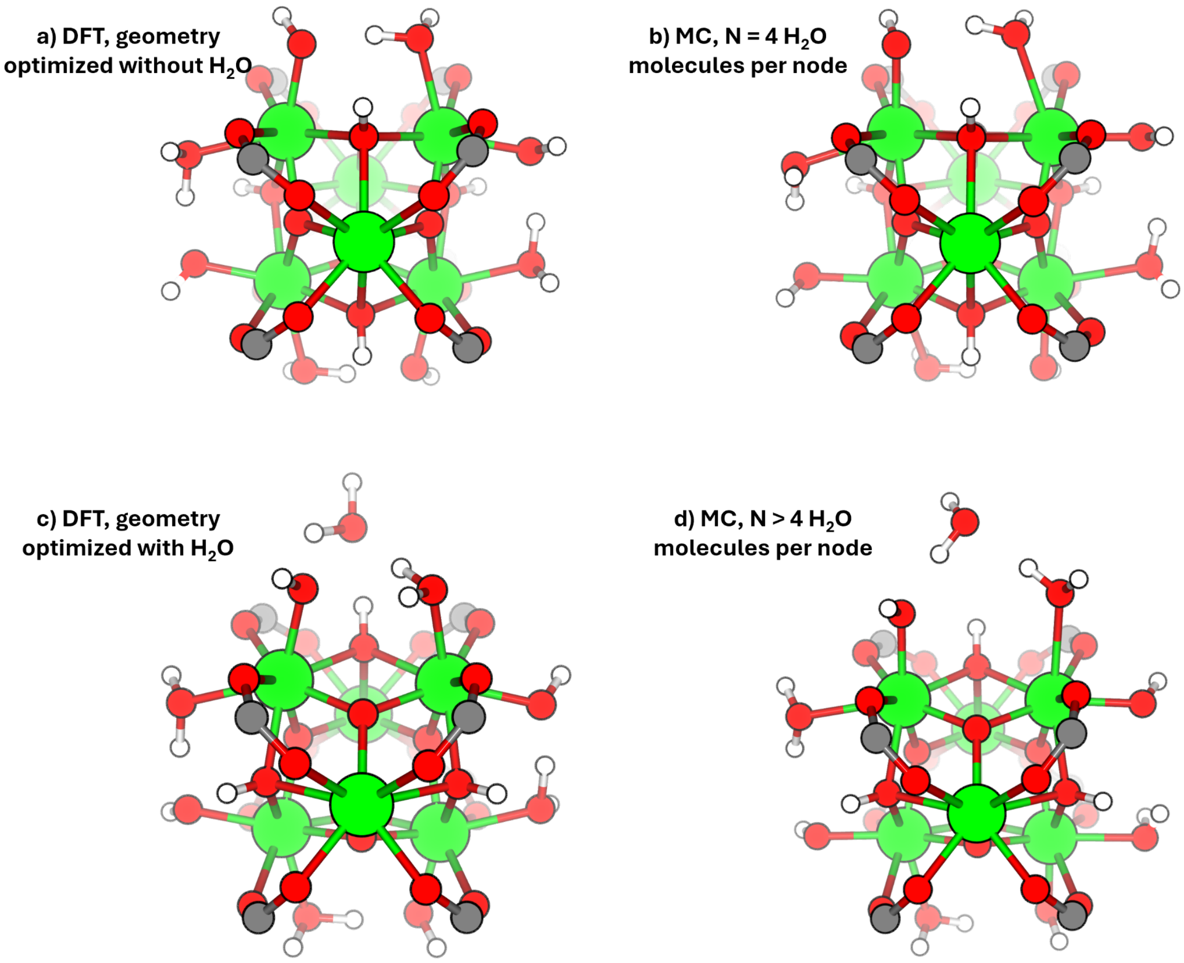

Figure S4. *Comparison of the DFT-optimized structures of the Zr node with a snapshot from the TMMC simulations for the OH (no terminal H_2O structure) reveals notable structural adjustments. In the absence of nonbonded water molecules, the simulation shows that the node forms a bond between terminal oxo and aquo ligands (b), similar to the DFT-optimized structure (a). Furthermore, when another molecule is present near this site, we observe a rotation of the terminal aquo ligand (d), mirroring the DFT optimization results (c).*

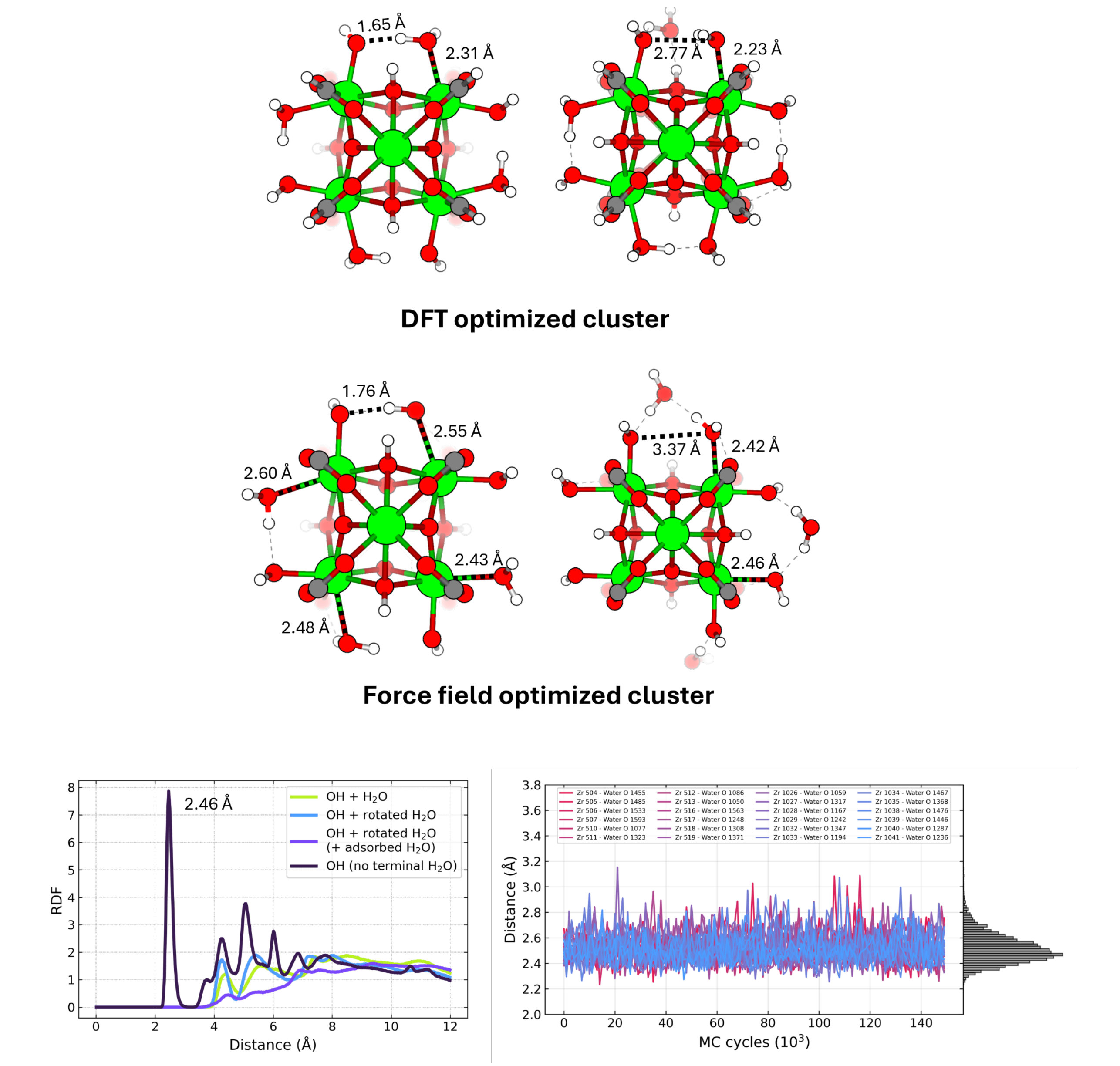

Figure S5. *DFT- and force field-optimized clusters (DFT: top, force field for OH (no terminal H_2O structure: middle) with specific distances highlighted. In the force field-optimized structure, only the adsorbed water was optimized, while the rest remained rigid, consistent with the protocol used in MC simulations. Bottom left: the RDF of oxygen atoms in adsorbed water relative to Zr atoms. Only the OH (no terminal H_2O) structure shows a strong peak at approximately 2.46 Å, indicating close contact that mimics a coordination bond. Bottom right: the distance between Zr atoms and the oxygen atoms of adsorbed water molecules (6×4 = 24, representing four water molecules on four Zr sites per node, with six nodes in the simulation unit cell). This demonstrates that the water molecules retain their position near Zr, validating that the model accurately mimics coordination bonds, which remain intact during the simulation.*

Table S2. *Adsorption energies (host-guest energies) from force-field-based geometry optimization of a single water molecule within a rigid framework in RASPA.*

	minimized adsorption energy (kJ/mol)
OH + H_2O	−43.2
OH + rotated H_2O	−71.9
OH + rotated H_2O (+ adsorbed H_2O)	−38.7
OH (no terminal H_2O)	−94.5

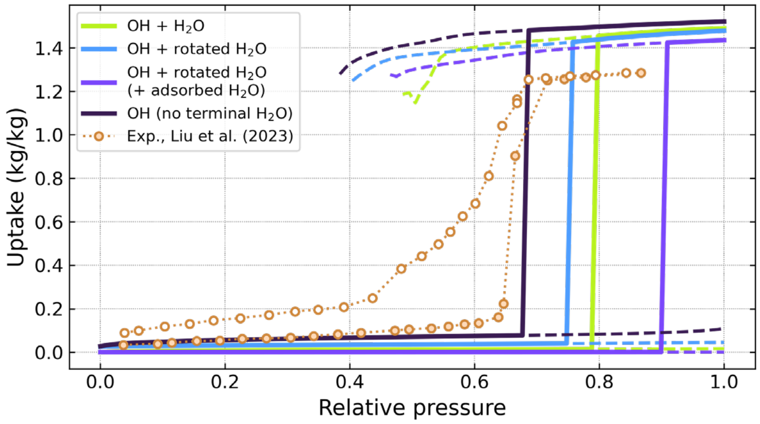

Figure S6. *TMMC isotherms with metastable branches depicted as dashed lines. The metastable branches represent the limits of phase stability, indicating that the hysteresis loop should remain confined between them. Note that the spinodal of the low-density phase (limit of stability) extends beyond the bulk saturation pressure.*

References

(1) Liu, J.; Prelesnik, J. L.; Patel, R.; Kramar, B. V.; Wang, R.; Malliakas, C. D.; Chen, L. X.; Siepmann, J. I.; Hupp, J. T. A Nanocavitation Approach to Understanding Water Capture, Water Release, and Framework Physical Stability in Hierarchically Porous MOFs. *J Am Chem Soc* **2023**, *145* (51), 27975–27983. https://doi.org/10.1021/JACS.3C07624.

(2) Sarkisov, L.; Bueno-Perez, R.; Sutharson, M.; Fairen-Jimenez, D. Materials Informatics with PoreBlazer v4.0 and the CSD MOF Database. *Chemistry of Materials* **2020**, *32* (23), 9849–9867. https://doi.org/10.1021/ACS.CHEMMATER.0C03575.

(3) Siderius, D. W.; Shen, V. K. Use of the Grand Canonical Transition-Matrix Monte Carlo Method to Model Gas Adsorption in Porous Materials. *Journal of Physical Chemistry C* **2013**, *117* (11), 5861–5872. https://doi.org/10.1021/jp400480q.

(4) Shen, V. K.; Errington, J. R. Metastability and Instability in the Lennard-Jones Fluid Investigated by Transition-Matrix Monte Carlo. *Journal of Physical Chemistry B* **2004**, *108* (51), 19595–19606. https://doi.org/10.1021/jp040218y.

(5) Errington, J. R. Direct Calculation of Liquid–Vapor Phase Equilibria from Transition Matrix Monte Carlo Simulation. *J Chem Phys* **2003**, *118* (22), 9915–9925. https://doi.org/10.1063/1.1572463.

(6) Siderius, D. W.; Hatch, H. W.; Shen, V. K. Flat-Histogram Monte Carlo Simulation of Water Adsorption in Metal-Organic Frameworks. *Journal of Physical Chemistry B* **2024**, *128*, 4845. https://doi.org/10.1021/ACS.JPCB.4C00753

(7) Xie, H.; Atilgan, A.; Joodaki, F.; Cui, J.; Wang, X.; Chen, H.; Yang, L.; Zhang, X.; Son, F. A.; Idrees, K. B.; Wright, A. M.; Wells, J. L.; Morris, W.; Klein, J.; Franklin, L.; Harrington, F.; Herrington, S.; Han, S.; Kirlikovali, K. O.; Islamoglu, T.; Snurr, R. Q.; Farha, O. K. Hydrolytically Stable Metal–Organic Frameworks for Harvesting Water from Low Humidity Air. *submitted* **2024**.

(8) Zielkiewicz, J. Structural Properties of Water: Comparison of the SPC, SPCE, TIP4P, and TIP5P Models of Water. *Journal of Chemical Physics* **2005**, *123* (10), 104501. https://doi.org/10.1063/1.2018637/898431.
